# Main differences between two highly effective lipid-lowering therapies in subclasses of lipoproteins in patients with acute myocardial infarction

**DOI:** 10.1186/s12944-021-01559-w

**Published:** 2021-09-29

**Authors:** Leticia C. S. Pinto, Ana P. Q. Mello, Maria C. O. Izar, Nagila R. T. Damasceno, Antonio M. F. Neto, Carolina N. França, Adriano Caixeta, Henrique T. Bianco, Rui M. S. Póvoa, Flavio T. Moreira, Amanda S. F. Bacchin, Francisco A. Fonseca

**Affiliations:** 1 Escola Paulista de Medicina, Setor de Lípides, Aterosclerose e Biologia Vascular, Universidade Federal de São Paulo, UNIFESP, Rua Loefgren 1350, São Paulo, SP 04040-001 Brazil; 2 Faculdade de Saúde Pública, Universidade de São Paulo, USP, São Paulo, Brazil; 3 Instituto de Física, Universidade de São Paulo, USP, São Paulo, Brazil; 4 Universidade Santo Amaro, UNISA, São Paulo, Brazil

## Abstract

**Background:**

Large observational studies have shown that small, dense LDL subfractions are related to atherosclerotic cardiovascular disease. This study assessed the effects of two highly effective lipid-lowering therapies in the atherogenic subclasses of lipoproteins in subjects with ST-segment elevation myocardial infarction (STEMI).

**Methods:**

Patients of both sexes admitted with their first myocardial infarction and submitted to pharmacoinvasive strategy (*N* = 101) were included and randomized using a central computerized system to receive a daily dose of simvastatin 40 mg plus ezetimibe 10 mg or rosuvastatin 20 mg for 30 days. Intermediate-density lipoprotein (IDL) and low-density lipoprotein (LDL) subfractions were analysed by polyacrylamide gel electrophoresis (Lipoprint System) on the first (D1) and 30th days (D30) of lipid-lowering therapy. Changes in LDL and IDL subfractions between D1 and D30 were compared between the lipid-lowering therapies (Mann-Whitney U test).

**Results:**

The classic lipid profile was similar in both therapy arms at D1 and D30. At D30, the achievement of lipid goals was comparable between lipid-lowering therapies. Cholesterol content in atherogenic subclasses of LDL (*p* = 0.043) and IDL (*p* = 0.047) decreased more efficiently with simvastatin plus ezetimibe than with rosuvastatin.

**Conclusions:**

Lipid-lowering therapy with simvastatin plus ezetimibe was associated with a better pattern of lipoprotein subfractions than rosuvastatin monotherapy. This finding was noted despite similar effects in the classic lipid profile and may contribute to residual cardiovascular risk.

**Trial registration:**

ClinicalTrials.gov, NCT02428374, registered on 28/09/2014.

## Introduction

Highly effective statins alone or combined with ezetimibe have been recommended for high-risk patients, according to recent guidelines [1, 2]. The achievement of target LDL-C levels seems important for plaque stability and a decrease in recurrent events after acute coronary syndrome [3–5]. However, the pattern of lipoprotein subfractions after lipid-lowering therapies also seems important. Small LDL particles are common in patients with hypertriglyceridemia. After the metabolism of triglyceride-enriched particles by lipoprotein lipase, followed by exchanges of triglycerides and cholesterol with other lipoproteins, mediated by the cholesterol ester transfer protein, further delipidation by hepatic lipase occurs, and small and dense LDL particles are formed [6]. For the same amount of cholesterol, a higher number of LDL particles are present among patients with small LDL subfractions. Due to the lower affinity of these particles for LDL receptors in the liver, prolonged circulation time favours oxidation and uptake by endothelial cells, contributing to plaque formation [6]. Recently, in a cohort of subjects without prior cardiovascular disease conducted in Japan, small, dense LDL was associated with a higher risk of coronary heart disease [7]. Accordingly, a meta-analysis involving 21 studies and 30,628 individuals confirmed the positive relationship between small, dense LDL and ischaemic heart disease [8]. Furthermore, in China, a country with one of the highest mortality rates from stroke, small and dense subclasses of LDL particles were more prevalent in subjects with acute ischaemic stroke [9]. The pattern of LDL particles can be examined by various methods, such as nuclear magnetic resonance, gradient gel electrophoresis, and ultracentrifugation, and differences between these methods have been reported [10]. Recently, a new equation using variables of the standard lipid panel was proposed and validated in a large cohort [11].

The greater effectiveness of combining ezetimibe to achieve LDL-C targets in relation to doubling statin doses has been established [12], but their effects on lipoprotein subfractions have been less reported, with the majority of studies finding a decrease in cholesterol content without a shift in the more or less atherogenic subfractions [13–18]. Interestingly, rosuvastatin therapy was associated with faster catabolism of large buoyant LDL particles [19].

Finally, residual inflammatory risk may be reduced by lipid-lowering therapy [20]. However, additional effects on high-sensitivity C-reactive protein (hsCRP) levels by ezetimibe therapy in subjects under statin therapy remain controversial [21, 22].

This work assessed the effects of two highly effective lipid-lowering therapies on the pattern of atherogenic subclasses of lipoproteins in patients with ST-segment elevation acute myocardial infarction (STEMI). Highly effective lipid-lowering therapies were considered those prescribed with the aim of achieving a 50% or greater reduction in LDL-C [1, 2].

## Methods

### Study population

For this study, 101 consecutive patients of both sexes with their first myocardial infarction were included as part of the B And T Types of Lymphocyte Evaluation in Acute Myocardial Infarction (BATTLE-AMI study, NCT02428374) [23]. All patients underwent pharmacological thrombolysis in the first 6 h followed by coronary angiogram and percutaneous coronary intervention (PCI) when needed in the first 24 h of STEMI (pharmacoinvasive strategy). Patients with primary PCI were not included in this trial. The key exclusion criteria were clinical instability, use of lipid-lowering or immunosuppressant therapies, autoimmune disease, known malignancy, pregnancy or signs of active infections. After hospital admission, these patients were randomized to be treated with simvastatin 40 mg plus ezetimibe 10 mg qd (Vytorin®, MSD) or rosuvastatin 20 mg qd (Crestor®, AstraZeneca) using a central computerized system (battle-ami.huhsp.org.br).

### Laboratory assays

Laboratory analyses and coronary angiograms and PCI were performed in *Hospital São Paulo* (a tertiary universitary hospital). The lipoprotein subfraction analysis was assessed in *Faculdade de Saude Publica* (*Universidade de Sao Paulo – USP*).

Blood samples (20 mL) were collected on the same day of hospitalization or on the morning of the following day in case of overnight hospitalization (D1) and after 30 days of STEMI (D30) and were promptly stored at − 80 °C until lipoprotein analysis. IDL and LDL subfractions were classified and measured by the Lipoprint System (Quantimetrix Corporation, CA, USA) according to the manufacturer’s instructions [24, 25]. The LDL_1,2_ subfractions were classified as large, buoyant LDLs (lbLDL), and subfractions LDL_3, 4, 5, 6, 7_ were classified as smaller and denser particles. LDL subfractions were adjusted by serum total cholesterol and are expressed as mg/dL.

Routine laboratory assays were performed in the Central Laboratory of the university hospital. High-sensitivity C-reactive protein (hsCRP) was measured by immunonephelometry.

Estimation of small dense LDL (sdLDL) subfractions was also examined by the Sampson equation [11].

### Statistical analysis

Statistical analysis was performed using SPSS version 18 (Armonk, NY, USA). The normality of continuous variables was examined by the Kolmogorov–Smirnov test. Data with a normal distribution are expressed as the mean ± SD, and non-Gaussian variables are expressed as the median (interquartile range [IQR]). Comparisons between groups were made by Student’s t test or the Mann–Whitney U test, as appropriate. Correlations between methods for sdLDL subfractions were made by Spearman’s rank test. A convenience sample was used due to the lack of previous studies comparing these treatments. Significance was set at a *P* value < 0.05.

## Results

**Fig. 1:**
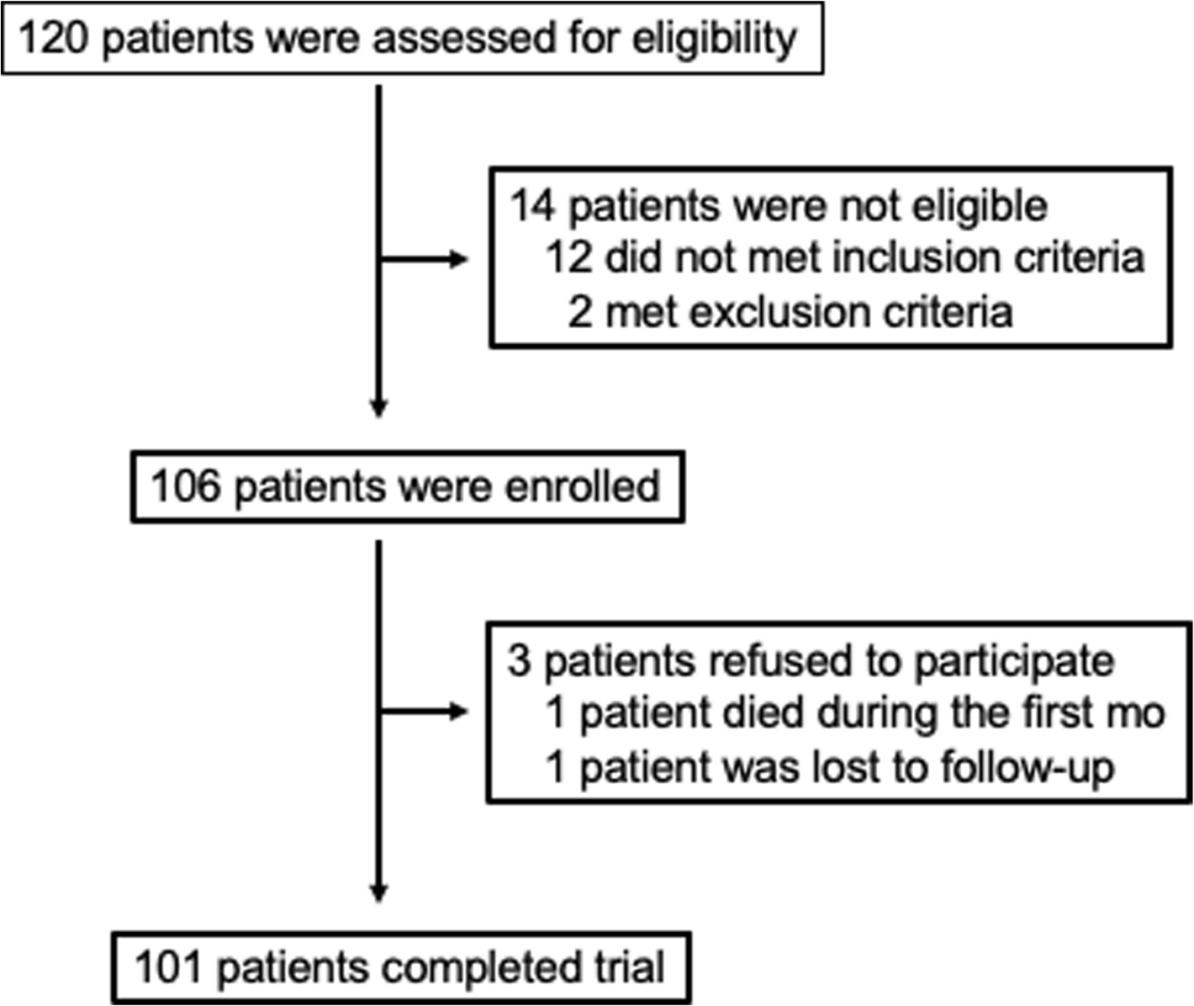
Flowchart. Only patients with a first myocardial infarction (STEMI) undergoing pharmacological thrombolysis during the first 6 h and coronary angiogram followed when necessary by percutaneous coronary intervention in the first 24 h were included. Patients receiving lipid-lowering or immunosuppressive therapy and those with clinical instability and contraindications or known intolerances for the lipid-lowering drugs of the study were excluded. One patient died during the first month after myocardial infarction

A total of 101 patients were included in the study (Fig. 1). The main clinical characteristics of the study population are shown in Table 1 and did not differ between groups. All patients had their first STEMI, and the proportion of culprit coronary artery and infarct size estimated by high-sensitivity troponin T (hsTNT) were similar in both groups.

**Table 1:** Clinical and laboratory characteristics of the study population

Parameter	Simvastatin plus ezetimibe *N* = 50	Rosuvastatin *N* = 51	*P value*
Age (years),	59 (52–65)	58 (55–64)	0.78
Males, n (%)	39 (76)	36 (72)	0.77
Smokers, n (%)	34 (67)	37 (74)	0.56
Hypertensives, n (%)	36 (71)	36 (72)	0.77
Diabetes, n (%)	16 (31)	19 (38)	0.62
BMI, kg/m2	26.9 (24.0–31.4)	25.3 (24.2–27.8)	0.47
HbA1c (%)	6.0 (5.5–6.7)	6.0 (5.6–6.7)	0.60
Glucose, mg/dL	129 (99–169)	121 (105–167)	0.78
hsTNT, ng/L	5881 (2273–12,231)	5985 (2409–10,020)	0.55
Creatinine, mg/dL	0.90 (0.79–1.11)	0.89 (0.77–1.00)	0.35
GFR, mL/min/m^2^	86 (70–93)	86 (73–98)	0.79
SBP, mm Hg	125 (110–137)	126 (111–137)	0.64
DBP, mm Hg	80 (67–83)	78 (71–89)	0.48
Culprit coronary artery			
Left anterior descending, n (%)	25 (50)	26 (51)	0.92
Right coronary artery, n (%)	18 (36)	19 (37)	0.90
Left circumflex artery, n (%)	7 (14)	6 (12)	0.74

Continuous variables are medians (IQRs). *LAD* left anterior descending artery, *RCA* right coronary artery, *LCX* left circumflex, *hsTNT* high-sensitivity troponin T. Categorical variables were compared by Pearson’s chi-square test, and continuous variables were compared by the Mann–Whitney U test

### Classic lipid profiles

Table 2 shows the classic lipid profiles at D1 and D30. Lipids were comparable at D1, and the effects of lipid-lowering therapies were similar at D30. The proportions of LDL-C and non-HDL-C targets achieved according to the European Society of Cardiology/European Atherosclerosis Society [1] were comparable between groups. LDL-C < 55 mg/dL was achieved by 57% of those patients in the simvastatin plus ezetimibe group and by 46% in the rosuvastatin group. For non-HDL-C < 85 mg/dL, 47 and 51% of patients achieved these goals in the simvastatin plus ezetimibe and rosuvastatin groups, respectively. The achievement of LDL-C < 70 mg/dL, suggested by the American College of Cardiology/American Heart Association [2], was observed in 67% of patients in the simvastatin plus ezetimibe group and in 60% of subjects treated with rosuvastatin (*P* = 0.51, Pearson chi-square test).

**Table 2:** Classic lipid profile at D1 and D30 by groups of lipid-lowering therapies

Parameters	Simvastatin plus ezetimibe *N* = 50	Rosuvastatin monotherapy *N* = 51	*P* value
At baseline (D1)			
Cholesterol, mg/dL	188 (174–223)	199 (168–227)	0.83
LDL-C, mg/dL	127 (113–153)	129 (107–150)	0.73
HDL-C, mg/dL	40 (31–46)	36 (31–45)	0.32
Triglycerides, mg/dL	140 (98–214)	157 (98–232)	0.42
Non-HDL-C, mg/dL	154 (137–193)	160 (139–193)	0.92
After 30 days (D30)			
Cholesterol, mg/dL	121 (98–141)	119 (105–141)	0.53
LDL-C, mg/dL	58 (43–82)	61 (50–82)	0.39
HDL-C, mg/dL	37 (31–44)	37 (32–40)	0.93
Triglycerides, mg/dL	133 (107–187)	140 (100–178)	0.93
Non-HDL-C, mg/dL	78 (65–112)	83 (70–105)	0.66

Values are medians (IQRs). Blood samples were collected on the first (D1) and 30 (D30) days after myocardial infarction. Variables were compared by the Mann–Whitney U test

### Subfractions of intermediate-density lipoproteins (IDL)

**Fig. 2:**
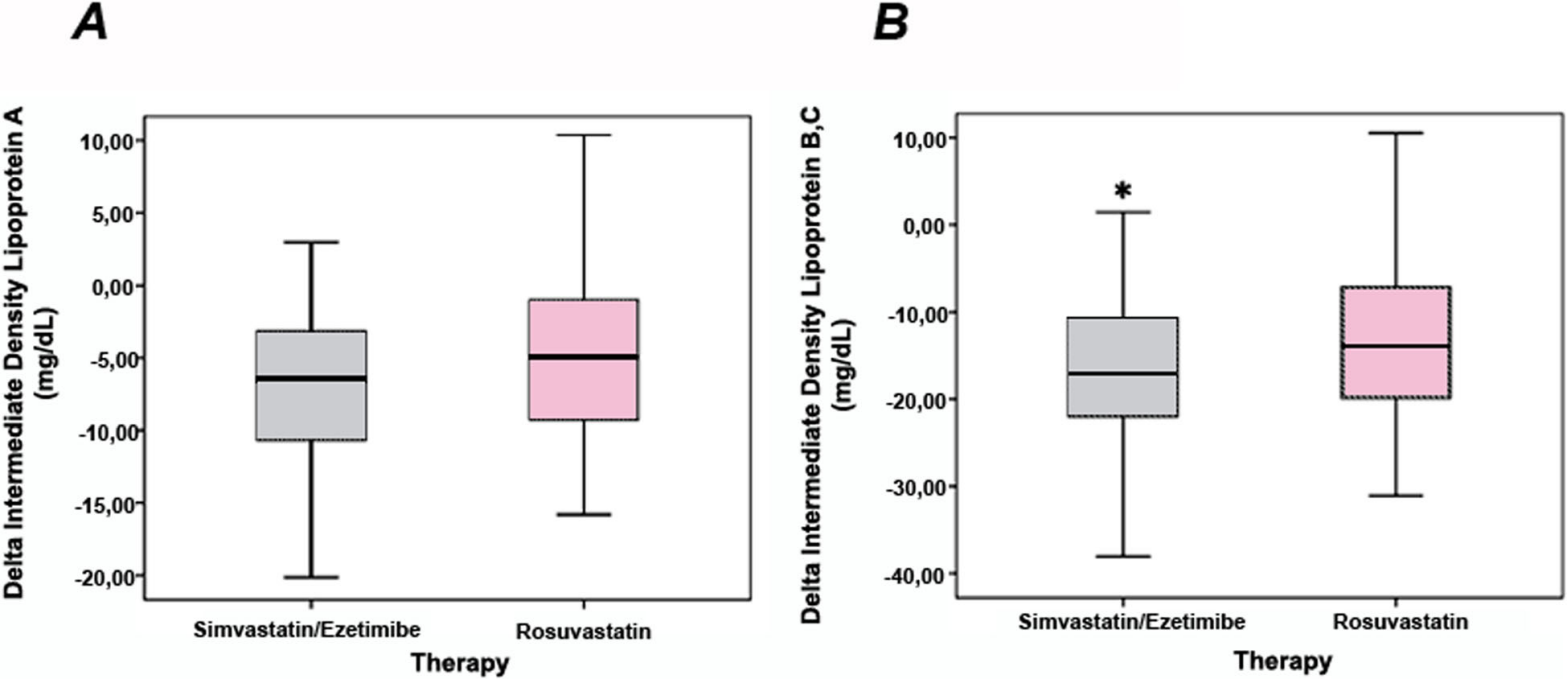
Delta (final minus initial content in cholesterol) of intermediate-density lipoprotein (IDL) subfractions by lipid-lowering therapies. **A** The delta of IDL-A (nonatherogenic) was similar after simvastatin plus ezetimibe or rosuvastatin treatment. **B** The delta of IDL-B plus IDL-C (atherogenic) showed higher effectiveness with simvastin plus ezetimibe than rosuvastatin monotherapy (*P = 0.047, Mann–Whitney U test)

Non-atherogenic (IDL-A) and atherogenic subfractions of intermediate-density lipoproteins (IDL-B and IDL-C) are shown in Table 3. At D1, similar levels of cholesterol content in nonatherogenic IDL-A were found in both groups, but the cholesterol titers from the atherogenic subfractions of intermediate-density lipoproteins (IDL-B plus IDL-C) were higher in the simvastatin plus ezetimibe group. To examine the effects of therapies, delta analysis was performed. After 30 days of lipid-lowering therapy, no significant differences in subfractions of IDL were found, but patients treated with simvastatin plus ezetimibe more efficiently decreased the cholesterol content in the atherogenic IDL subclasses (Delta, *P* = 0.047 vs. rosuvastatin group, Mann–Whitney U test), whereas no differences were observed between groups for cholesterol from the nonatherogenic IDL-A (Fig. 2).

**Table 3:** Cholesterol content in subfractions of intermediate- and low-density lipoproteins at baseline and after lipid-lowering therapy

Lipoprotein subfractions	Simvastatin plus ezetimibe *N* = 50	Rosuvastatin monotherapy *N* = 51	*P* value
At baseline (D1)			
IDL-A	16.0 (10.1–21.4)	12.5 (8.5–17.8)	0.06
IDL-B + C	40.1 (31.9–49.4)	35.2 (24.6–43.9)	0.02
LDL-1 + 2	50.4 (39.2–63.2)	52.5 (41.3–64.2)	0.38
LDL-3 + 4 + 5 + 6 + 7	3.6 (0.0–9.9)	2.8 (0.0–5.7)	0.09
After 30 days (D30)			
IDL-A	8.8 (6.8–12.5)	7.5 (5.8–10.7)	0.15
IDL-B + C	22.0 (18.7–29.2)	20.6 (17.5–27.0)	0.21
LDL-1 + 2	25.0 (20.0–33.1)	24.8 (18.7–34.3)	0.96
LDL-3 + 4 + 5 + 6 + 7	1.8 (0.0–4.5)	2.0 (0.0–6.0)	0.75

Values are medians (IQRs) of cholesterol from subfractions of intermediate-density lipoproteins (IDL) and low-density lipoproteins (LDL). IDL-A is considered nonatherogenic, while IDL-B and IDL-C are atherogenic. LDL-1 and LDL-2 are large and buoyant (nonatherogenic), while LDL-3 to LDL-7 are considered atherogenic. Lipoprotein subfractions were adjusted by total serum cholesterol and are expressed in mg/dL. Data were compared by the Mann–Whitney U test

### Subfractions of low-density lipoprotein (LDL)

**Fig. 3:**
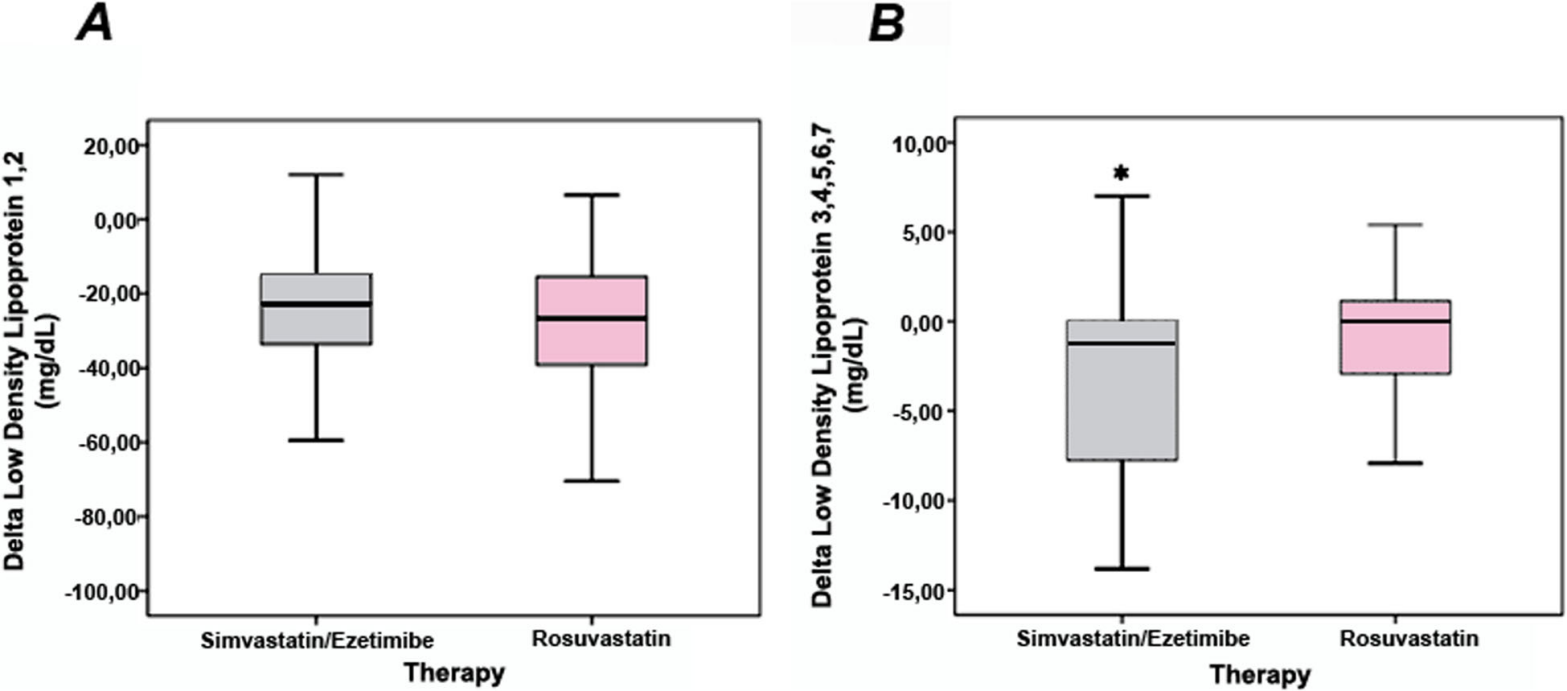
Delta (final minus initial cholesterol content) in low-density lipoprotein (LDL) subfractions by lipid-lowering therapies. **A** The delta of LDL-1 plus LDL-2 (nonatherogenic) was similar after exposure to lipid-lowering therapies. **B** The delta of atherogenic LDL subfractions (LDL-3 to LDL-7) showed greater reduction by simvastatin plus ezetimibe in comparison with rosuvastatin (*P = 0.043, Mann–Whitney U test)

Table 3 shows the cholesterol content of the LDL subfractions. At D1, both lipid-lowering groups had similar cholesterol concentrations of nonatherogenic LDL (LDL-1 and LDL-2) and atherogenic LDL subfractions (LDL-3 to LDL-7). At D30, no significant differences were observed for atherogenic and nonatherogenic LDL particles (Table 3), but simvastatin plus ezetimibe more efficiently decreased cholesterol content in the atherogenic subfractions (Delta, *P* = 0.043, Mann–Whitney U test), whereas a similar effect was observed for the nonatherogenic LDL subfractions (Fig. 3).

Small, dense LDL (sdLDL) and large, buoyant LDL (lbLDL) particles were also examined by the Sampson equation (Table 4). No differences at baseline or after lipid-lowering treatments were found. However, lbLDL-C estimated by the Sampson equation was correlated with LDL_1,2_ (less atherogenic) obtained by the lipoprint system at baseline (rho = 0.43, *P* < 0.001) and at 30 days (rho = 0.58, *P* < 0.001). In addition, sdLDL (Sampson equation) and LDL_3–7_ (lipoprint system) were also correlated at baseline (rho = 0.29, *P* = 0.004) and at D30 (rho = 0.40, *P* < 0.001).

**Table 4:** Cholesterol content in subfractions of lipoproteins at baseline and after lipid-lowering therapy by the Sampson equation

Lipoproteins	Simvastatin plus ezetimibe *N* = 50	Rosuvastatin monotherapy *N* = 51	*P* value
At baseline (D1)			
LDL-C	124 (111–154)	127 (101–147)	0.58
VLDL-C	24 (17–38)	27 (17–43)	0.55
lbLDL-C	88 (69–110)	84 (62–99)	0.32
sdLDL-C	41 (33–50)	42 (31–55)	0.94
After 30 days (D30)			
LDL-C	54 (39–76)	58 (46–80)	0.23
VLDL-C	19 (15–27)	21 (15–28)	0.58
lbLDL-C	31 (21–45)	36 (26–49)	0.18
sdLDL-C	23 (17–30)	23 (20–30)	0.36

Values (mg/dL) are medians (IQRs) of cholesterol from subfractions of large and buoyant (lbLDL) or small and dense (sdLDL) lipoproteins obtained at baseline and after 30 days based on the Sampson equation from the standard lipid panel. Data were compared by the Mann–Whitney U test

### High-sensitivity C-reactive protein

The levels of hsCRP at D1 (median [IQR]) were similar in both lipid-lowering treatment groups (25.6 [12.4–47.8] vs. 21.1 [10.2–46.9] mg/L, *P* = 0.45, Mann–Whitney U test) for the simvastatin plus ezetimibe and rosuvastatin groups, respectively. After 30 days, substantial and comparable decreases were found in both groups (2.14 [0.90–6.27] vs. 1.92 [0.95–4.72] mg/L, *P* = 0.78, Mann–Whitney U test) for the simvastatin plus ezetimibe and rosuvastatin groups, respectively. The delta of hsCRP titers (D30 – D1) observed after treatment was also similar between groups of lipid-lowering therapies (*P* = 0.91, Mann–Whitney U test). In addition, at D30, the achievement of hsCRP levels < 2 mg/L was similar between the lipid-lowering groups (44% vs. 50%, *P* = 0.56, Pearson chi-square test) for the simvastatin plus ezetimibe and rosuvastatin groups. The achievement of both goals (LDL-C < 70 mg/dL and hsCRP levels < 2 mg/L) was observed in 29 and 28% of patients treated with simvastatin plus ezetimibe and rosuvastatin, respectively (*P* = 0.97, Pearson chi-square test).

## Discussion

This study compared two highly effective lipid-lowering therapies in patients with STEMI and revealed that simvastatin plus ezetimibe more efficiently decreased the atherogenic subfractions of intermediate- and low-density lipoproteins than rosuvastatin monotherapy. Despite the strong evidence favouring statin monotherapy as a first option for lipid-lowering therapy in subjects at very high cardiovascular risk, the need for additional hypolipidaemic drugs is frequently necessary to achieve current LDL-C and non-HDL-C targets. In addition, simvastatin plus ezetimibe produced a similar reduction in hsCRP levels, a useful inflammatory biomarker with prognostic implications after acute myocardial infarction [26, 27]. However, approximately half of the patients in both arms of lipid-lowering treatment remained with hsCRP levels above 2 mg/L, suggesting the need for additional therapy to reduce the inflammatory residual risk.

**Fig. 4:**
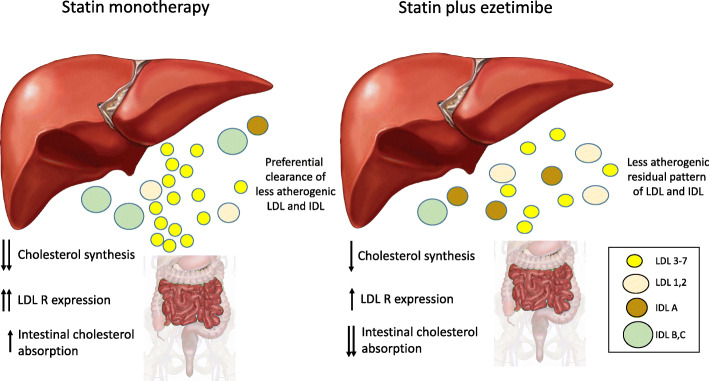
Effects of lipid-lowering therapies on the atherogenic and nonatherogenic subfractions of lipoproteins. Rosuvastatin monotherapy decreased endogenous cholesterol synthesis and augmented both LDL receptor (LDL-R) expression and intestinal cholesterol absorption. The higher expression of LDL-R facilitated the clearance of large and buoyant LDL particles, which had more affinity to LDL-R than small dense particles. Following the use of simvastatin plus ezetimibe, other mechanisms were involved, and the decrease in cholesterol absorption by ezetimibe increased cholesterol synthesis and LDL-R expression. Differences in LDLR expression and lower intestinal cholesterol absorption may contribute to differences in the residual lipoprotein subfraction pattern. Higher effectiveness in the clearance of atherogenic IDL particles was also observed following simvastatin plus ezetimibe therapy compared with rosuvastatin monotherapy

Large observational studies have shown that small, dense LDL has been related to coronary heart disease [28–30]. The lower affinity of the small, dense LDL to LDL receptor results in a longer plasma resident time, increasing the opportunity for LDL oxidation and uptake by the vessel wall [6]. In the Justification for the Use of Statins in Prevention: an Intervention Trial Evaluating Rosuvastatin (JUPITER), rosuvastatin treatment was associated with smaller reductions in the small LDL subfractions than in the larger and buoyant subfractions [31]. Furthermore, incident cardiovascular disease was not associated with baseline LDL-C but with atherogenic non-HDL subfractions, suggesting a residual cholesterol risk [31]. In fact, by decreasing endogenous cholesterol synthesis, statins increase LDL receptor expression in the liver, promoting higher uptake of LDL particles, mainly those large and buoyant particles, which have higher affinity for the LDL receptor, explaining our findings (Fig. 3). Recently, lipid-lowering treatment with a monoclonal antibody against proprotein convertase subtilisin/kexin type 9 (PCSK9), which markedly increases the expression of the LDL receptor in the liver, resulted in a greater decrease in the large and buoyant LDL subfractions than in the smaller LDL subfractions [32]. Bays et al. examined the effects of atorvastatin 40 mg when doubling the dose or combining it with ezetimibe among high-risk patients, and they found with both therapies that LDL particle pattern A (considered less atherogenic) was the lipoprotein predominantly cleared [33]. Together, these studies suggest the greatest effect in reducing large and buoyant LDL particles through the greatest affinity of these particles with LDL receptors, mainly following potent statins or PCSK9 inhibitors. Ezetimibe also increases the expression of LDL receptors, but possibly to a lesser magnitude [34] (Fig. 4).

The results obtained for the lipoprotein subfraction assessment using the Lipoprint system were correlated to the Sampson equation; however, using this last methodology, no significant differences between lipid-lowering therapies were found. Discrepancies between methods evaluating LDL subfractions have been previously reported [10]. In addition, important differences between the effects of highly effective lipid-lowering therapies on several lipids, examined by lipidomics, were recently reported, suggesting that small differences in the subfractions of LDL may be counterbalanced by favourable effects on other lipid classes, such as ceramides [35].

In our study, most atherogenic subfractions of intermediate-density lipoproteins (ILD B and IDL C) were also more effectively reduced by simvastatin plus ezetimibe therapy than with rosuvastatin monotherapy. These subfractions of intermediate-density lipoproteins can contribute to residual cholesterol risk, as shown in the JUPITER study [31]. Thus, decreasing non-HDL cholesterol by combined therapy not only increases the achievement of lipid targets but also seems to improve the pattern of lipoprotein subfractions. Thus, by similarly decreasing LDL and non-HDL cholesterol levels but with lower dependency of LDL receptors in the liver, the use of ezetimibe promoted a less atherogenic residual pattern of lipoproteins.

Recently, a machine learning-based clustering study among patients with STEMI under statin therapy evaluated a lipid panel composed of apo A1, Apo B, HDL-C, triglycerides, LDL-C, total cholesterol and lipoprotein (a) [Lp (a)]. The study revealed that the cluster with high levels of Lp (a) and low levels of apoA1 and HDL-C identified those patients with the highest recurrent events and mortality [36].

Even without a causal role in cardiovascular disease, hsCRP levels have been related to long-term prognosis after myocardial infarction [37], and the achievement of dual goals (LDL-C and hsCRP) has identified patients with better event-free survival in the Pravastatin or Atorvastatin Evaluation and Infection Therapy trial (PROVE-IT) [38] and the Improved Reduction of Outcomes: Vytorin Efficacy International Trial (IMPROVE-IT) [21] trials. In our study, the achievement of both targets (LDL-C < 70 mg/dL and hsCRP< 2 mg/L) was reported in a similar proportion of patients treated with simvastatin plus ezetimibe or rosuvastatin. However, the achievement of these goals was observed by less than approximately one-third of these patients. Thus, the need for additional lipid-lowering and anti-inflammatory therapy seems necessary to reduce the inflammatory residual risk, as has been more recently demonstrated [38–43]. Finally, combined therapy to achieve cholesterol goals using multiple mechanisms appears to be safer in the long term of intensive lipid-lowering therapy, allowing exposure to lower statin doses and reducing pharmacokinetic interactions and adverse muscle events [44].

### Comparisons with other studies and what the current work adds to the existing knowledge

This study compared a potent statin with a moderately potent statin combined with ezetimibe in lipoprotein subclasses and revealed differences in lipoprotein subfractions between these lipid-lowering strategies. This head-to-head randomized comparison adds new information to previous studies reporting the effects of potent statins alone [31] or combined with ezetimibe [33].

### Strengths and limitations

This study shows a direct comparison between two lipid-lowering therapies aimed at achieving 50% or more LDL-C reduction and reveals important aspects. First, there were some differences between treatments in the LDL subfractions, despite similar standard lipid panels. Second, some goals were not achieved despite the use of recommended lipid-lowering therapies, particularly among very high-risk patients [1, 2]. The number of patients was relatively small but included only patients with STEMI under a pharmacoinvasive strategy, with comparable baseline clinical and laboratory characteristics, including troponin levels. Despite small differences between lipid-lowering therapies, these findings are important due to the direct comparisons between these lipid-lowering therapies after myocardial infarction, a period of high rates of atherosclerotic recurrent events.

## Conclusion

Lipid-lowering therapy with simvastatin plus ezetimibe was associated with better effects on the atherogenic LDL and IDL subfractions than rosuvastatin monotherapy. These differences were noted despite similar effects in the classic lipid profile and may contribute to residual cardiovascular risk.

### What are the clinical relevance and future perspectives?

The findings of the study suggest that a better pattern of LDL subfractions can be obtained by the combination of ezetimibe with a statin of moderate potency in comparison to a more potent statin. This result shows that statins combined with ezetimibe seem important not only for achieving lipid targets but also for better LDL subfraction patterns.

## Data Availability

The analysed database is available from the corresponding author upon request.

## Abbreviations

LDL: Low-density lipoprotein
IDL: Intermediate-density lipoprotein
hsCRP: high-sensitivity C-reactive protein
STEMI: ST-segment elevation acute myocardial infarction
PCI: Percutaneous coronary intervention
lbLDL: Large buoyant LDL
sdLDL: Small dense LDL
hsTNT: high-sensitivity troponin T
PCSK9: Proprotein convertase subtilisin/kexin type 9
Lp (a): Lipoprotein (a)
